# Supplementary material for: Molecular fingerprinting of biological nanoparticles with a label-free optofluidic platform
Source: Nat Commun. 2024 May 15;15:4109. doi: 10.1038/s41467-024-48132-4 (PMC11096335; doi:10.1038/s41467-024-48132-4)
Supplement: Supplementary file 1 — Supplementary Information [file 41467_2024_48132_MOESM1_ESM.pdf]

Molecular fingerprinting of biological nanoparticles with a label-free optofluidic platform

Alexia Stollmann,[1] Jose Garcia-Guirado,[1] Jae-Sang Hong,[2] Pascal Rüedi,[1] Hyungsoon Im,[2,3] Hakho Lee,[2,3] Jaime Ortega Arroyo,[1] Romain Quidant[1]**

AFFILIATIONS:

[1] Nanophotonic Systems Laboratory, Department of Mechanical and Process Engineering, ETH Zurich, 8092 Zurich, Switzerland

[2] Center for Systems Biology, Massachusetts General Hospital, Boston, Massachusetts 02114, United States

[3] Department of Radiology, Massachusetts General Hospital, Boston, Massachusetts 02114, United States

Correspondence to: jarroyo@ethz.ch , rquidant@ethz.ch

This PDF file includes:

Supplementary Figures 1 to 7

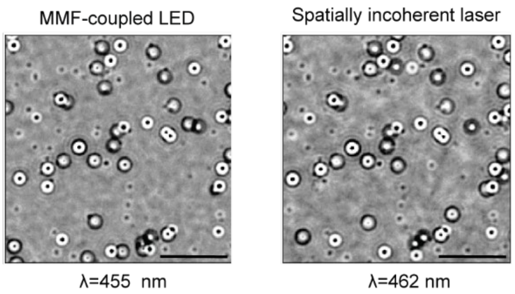

Supplementary Figure 1. Equivalence between spatially incoherent illumination schemes. Representative zoom-in images of the same sample comparing two different light sources used for producing spatially incoherent illumination. Both illumination schemes use the same MMF fibre. The spatial coherence of the laser source is drastically reduced upon passing through a rotating ground glass diffuser prior to coupling to the MMF fibre. Scale bars: 5 µm.

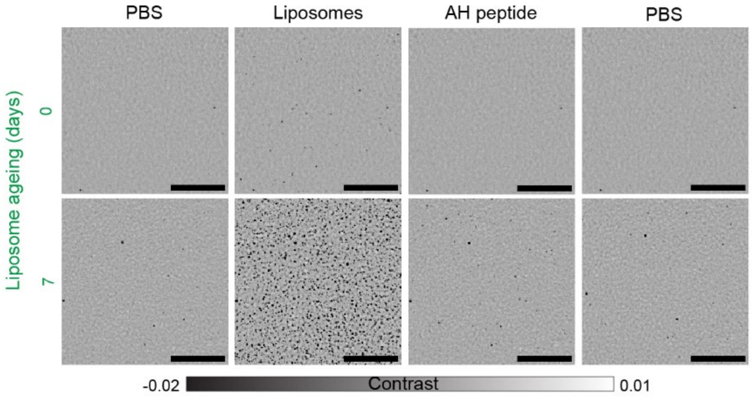

Supplementary Figure 2. Effect of liposome ageing on SLB formation. Liposome ageing affects the probability of spontaneous vesicle rupture but not the overall SLB quality upon treatment with the peptide. Representative zoom-in images for each preparation method at the different stages of the peptide-mediated supported lipid bilayer formation process. Scale bars: 5 µm.

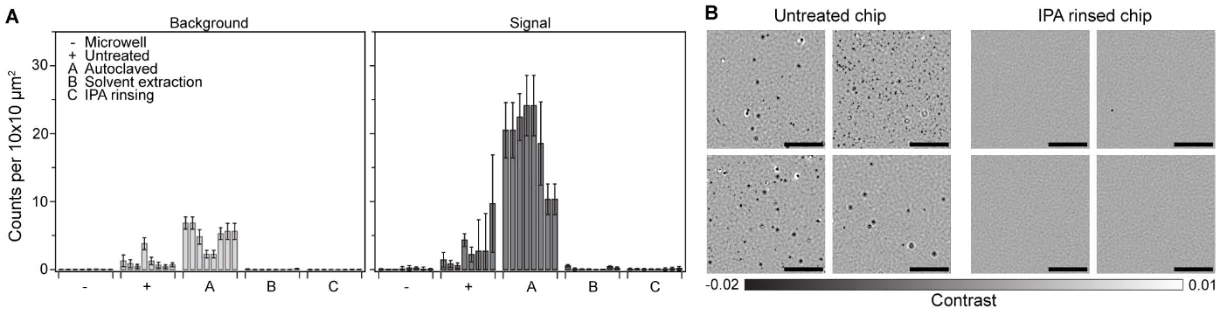

Supplementary Figure 3. Effect of different PDMS cleaning strategies to remove uncured oligomers. (A) Mean substrate defect densities as a function of different PDMS cleaning strategies. Microwells acted as negative control due to the lower PDMS surface area and thus lower likelihood for uncured oligomers to leach towards the substrate surface. Untreated PDMS chips were considered as a positive control. Bars represent the median defect density recorded within a single FOV over a sensing area of approximately 0.2 mm^2. Error bars represent ± SD over N independent FOVs (N = 20). Each box per category corresponds to an independent channel replica (M = 8). **(B)** representative zoom-in images of the substrate upon addition of PBS for an untreated and IPA rinsed PDMS chip, respectively. Scale bars: 10 µm.

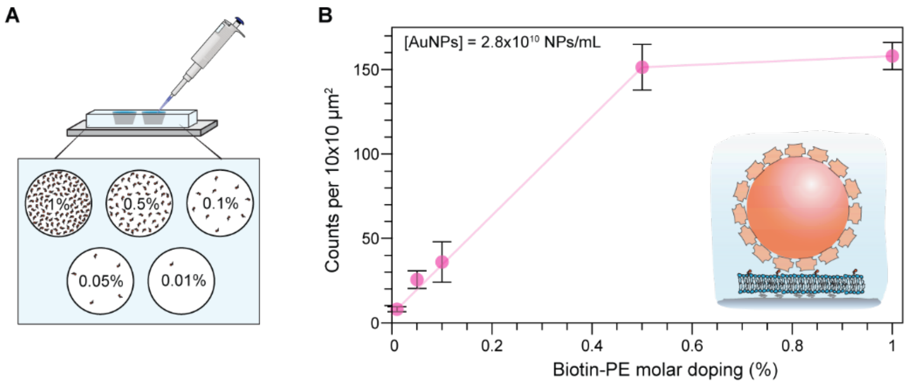

Supplementary Figure 4. Lipid composition of the SLB determines the capture site density. **(A)** Cartoon depicting the control over the capture sites by tuning the percentage of biotin-PE lipid included in the SLB formation. **(B)** Biotin-DOPE doping response curve for a fixed AuNP-SAv concentration. Data are expressed as mean ± SD over N independent FOVs (N = 102). Inset depicts the pulldown assay consisting of a biotinylated SLB and AuNP-SAv.

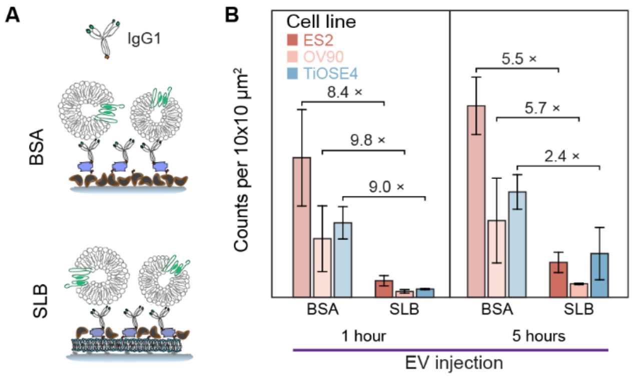

Supplementary Figure 5. Non-specific binding comparison between BSA and SLB. (A) Cartoon depicting the two surface functionalisation strategies compared using IgG1 isotype control antibody as the capture probe. **(B)** Number of non-specifically bound EVs for the two functionalisation schemes across three different ovarian cell lines. Numbers above the bars indicate the fold increase of non-specific binding to BSA functionalised chips compared to SLB ones. Errors bars indicate ± SD (N = 2).

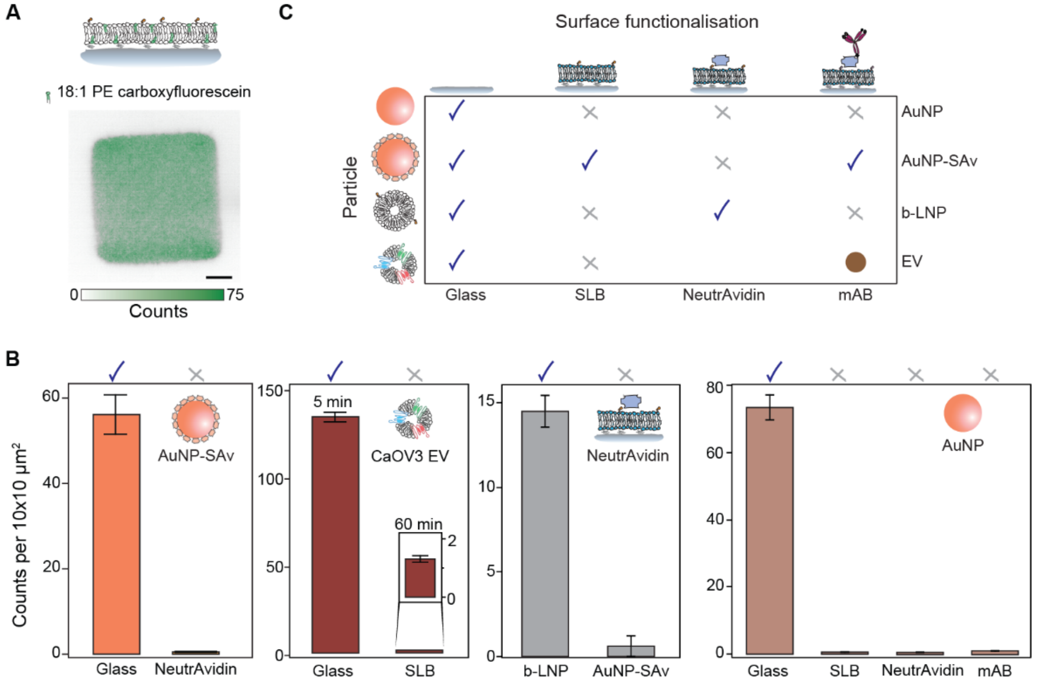

Supplementary Figure 6. Additional surface functionalisation validation. (A) Representative fluorescence image of a formed SLB 0.1% molar doped with a fluorescently-labelled lipid. The homogeneous fluorescence signal shows that surface defects are not resolvable under this imaging modality. Scale bar: 10 μm. **(B)** Average number of bound particles within a single FOV (66×66 μm^2) in paired positive (check mark) and negative control (cross) assays to validate different functionalisation steps. Data are expressed as mean ± SD over N independent FOVs (N = 20). **(C)** Tabulated schematic outlining all the positive and negative control assays (Fig. 4C, Supplementary

Fig. 6B, Fig. 6) that validate successful surface functionalisation. Check mark: positive control, cross: negative control, circle: target assay.

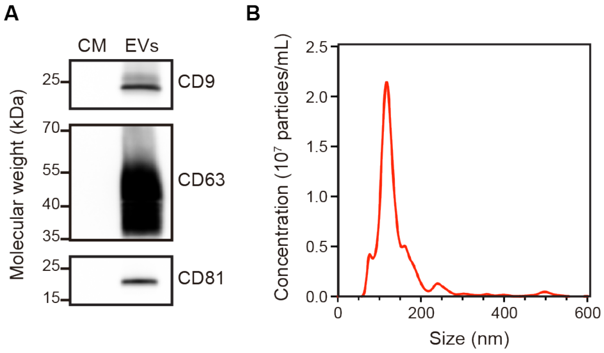

Supplementary Figure 7. ES2 extracellular vesicle characterisation. (A) Western blot analysis against the characteristic EV tetraspanin markers: CD9, CD63, CD81. CM: cultured medium. **(B)** Size and concentration determination via nanoparticle tracking analysis.
