# Supplementary material for: Molecular fingerprinting of biological nanoparticles with a label-free optofluidic platform
Source: Nat Commun. 2024 May 15;15:4109. doi: 10.1038/s41467-024-48132-4 (PMC11096335; doi:10.1038/s41467-024-48132-4)
Supplement: Supplementary file 4 — Reporting Summary [file 41467_2024_48132_MOESM4_ESM.pdf]

nature portfolio

Corresponding author(s): Jaime Ortega Arroyo

Last updated by author(s): Apr 8, 2024

Reporting Summary

Nature Portfolio wishes to improve the reproducibility of the work that we publish. This form provides structure for consistency and transparency in reporting. For further information on Nature Portfolio policies, see our Editorial Policies and the Editorial Policy Checklist.

Statistics

For all statistical analyses, confirm that the following items are present in the figure legend, table legend, main text, or Methods section.

n/a	Confirmed	
☐	☒	The exact sample size (*n*) for each experimental group/condition, given as a discrete number and unit of measurement
☐	☒	A statement on whether measurements were taken from distinct samples or whether the same sample was measured repeatedly
☐	☒	The statistical test(s) used AND whether they are one- or two-sided<br>*Only common tests should be described solely by name; describe more complex techniques in the Methods section.*
☒	☐	A description of all covariates tested
☒	☐	A description of any assumptions or corrections, such as tests of normality and adjustment for multiple comparisons
☐	☒	A full description of the statistical parameters including central tendency (e.g. means) or other basic estimates (e.g. regression coefficient) AND variation (e.g. standard deviation) or associated estimates of uncertainty (e.g. confidence intervals)
☐	☒	For null hypothesis testing, the test statistic (e.g. *F*, *t*, *r*) with confidence intervals, effect sizes, degrees of freedom and *P* value noted<br>*Give P values as exact values whenever suitable.*
☒	☐	For Bayesian analysis, information on the choice of priors and Markov chain Monte Carlo settings
☒	☐	For hierarchical and complex designs, identification of the appropriate level for tests and full reporting of outcomes
☒	☐	Estimates of effect sizes (e.g. Cohen's *d*, Pearson's *r*), indicating how they were calculated

Our web collection on statistics for biologists contains articles on many of the points above.

Software and code

Policy information about availability of computer code

Data collection	Data were collected using a custom-acquisition code that interfaces different hardware devices (Hamamatsu sCMOS camera, MadCityLabs motorized XY stage, MadCityLabs piezo Z stage, Thorlabs LED, NI FPGA) with the computer and allows real-time user interaction. Code was developed in LabVIEW 2020 using the propietary DLL functions provided by each of the hardware suppliers in their corresponding SDK. Importantly, the GUI interface is not central to the study described here, and any video acquisition software interfaced to the sample stage controller can be used; for instance: MicroManager, MaTLAB, or pySCAT.
Data analysis	LabVIEW 2020 was used in the initial data analysis pipeline to localise all individual single particle signals. Here established routines of PSF segmentation and particle localisation (radial symmetry centres) were used as detailed in the methods section. Subsequent analysis of the number of localisation was performed on Mathematica 12. The particle localisation and downsteam data analysis code are not central to the study described here as any single particle localisation software adapted to work for interferometric microscopy can be used.

For manuscripts utilizing custom algorithms or software that are central to the research but not yet described in published literature, software must be made available to editors and reviewers. We strongly encourage code deposition in a community repository (e.g. GitHub). See the Nature Portfolio guidelines for submitting code & software for further information.

Data

Policy information about availability of data

All manuscripts must include a data availability statement. This statement should provide the following information, where applicable:

- Accession codes, unique identifiers, or web links for publicly available datasets
- A description of any restrictions on data availability
- For clinical datasets or third party data, please ensure that the statement adheres to our policy

Source data are provided with this paper. The main data supporting the results in this study are available within the paper and its Supplementary Information. The raw and analysed datasets generated during the study are too large to be publicly shared yet are available for research purposes from the corresponding authors upon request. Requests will be fulfilled within 10 weeks.

Research involving human participants, their data, or biological material

Policy information about studies with human participants or human data. See also policy information about sex, gender (identity/presentation), and sexual orientation and race, ethnicity and racism.

Reporting on sex and gender	Not applicable. Study did not involve human participants.
Reporting on race, ethnicity, or other socially relevant groupings	Not applicable. Study did not involve human participants.
Population characteristics	Not applicable. Study did not involve human participants.
Recruitment	Not applicable. Study did not involve human participants.
Ethics oversight	Not applicable. Study did not involve human participants.

Note that full information on the approval of the study protocol must also be provided in the manuscript.

Field-specific reporting

Please select the one below that is the best fit for your research. If you are not sure, read the appropriate sections before making your selection.

☒ Life sciences ☐ Behavioural & social sciences ☐ Ecological, evolutionary & environmental sciences

For a reference copy of the document with all sections, see nature.com/documents/nr-reporting-summary-flat.pdf

Life sciences study design

All studies must disclose on these points even when the disclosure is negative.

Sample size	We measure individual biological nanoparticles using an immunnoaffinity pull-down assays. For each imaging scan of a non-negative control sample, a minimum of 10,000 particles are identified per scan to ensure robust statistics. The rationale for this number is individual particle statistics are governed by Poisson statistics. We obtain this minimum number of particles by controlling the density of capture sites and total sample area imaged, details of which are provided in the methods section. For each area scan a minimum of 20 FOVs are acquired.
Data exclusions	Chips showing defects in fabrication or unsuccessful surface functioanlisation were discarded from further downstream analysis. Unsuccessful surface functionalisation was determined by in-situ imaging and characterising the number of defects within the imaging chamber. Representative data is shown for each experiment.
Replication	A minimum of at least 3 chip replicates were performed for the molecular fingerprinting of different cell-line derived EV populations. All other experiments had a minimum of 3 chip replicates. In all cases, the number of replicates are stated in the corresponding figure captions.
Randomization	Not applicable. All measurements were by default performed on random samples of biological nanoparticles that are loaded into the microfluidic tubing. The solution loaded into the tubing was randomly selected from an eppendorf.
Blinding	Not applicable. All data were analysed by the same software using the same parameters, ensuing that the analysis is blind to the type of sample.

Reporting for specific materials, systems and methods

We require information from authors about some types of materials, experimental systems and methods used in many studies. Here, indicate whether each material, system or method listed is relevant to your study. If you are not sure if a list item applies to your research, read the appropriate section before selecting a response.

Materials & experimental systems

n/a	Involved in the study	
☐	☒	Antibodies
☐	☒	Eukaryotic cell lines
☒	☐	Palaeontology and archaeology
☒	☐	Animals and other organisms
☒	☐	Clinical data
☒	☐	Dual use research of concern
☒	☐	Plants

Methods

n/a	Involved in the study	
☒	☐	ChIP-seq
☒	☐	Flow cytometry
☒	☐	MRI-based neuroimaging

Antibodies

Antibodies used

Primary antibodies used in the immunoaffinity pull-down assays following the parameter convention:
name (functionalisation) (supplier, catalogue number, clone, lot number)

CD63 mAb (Biotin) (Ancell, Catalogue: 215-030, Clone: AHN16.1, Lot: 283502)
CD326 mAb (Biotin) (Ancell, Catalogue: 126-030, Clone: ABC8D4, Lot: 223303)
CD9 mAb (Biotin) (Ancell, Catalogue: 156-030, Clone: C3-3A2, Lot: 285004)
CD81 mAb (Biotin) (Ancell, Catalogue: 302-030, Clone: 1.3.3.22, Lot: 264803)
CA125 mAb (Biotin) (LSBio, Catalogue: LS-C86749, Clone: X52, Lot: 163723)
HE-4 mAb (Biotin) (LSBio, Catalogue: LS-C743705-50, Clone: 4B2-F10-E1, Lot: 222516)
Mouse IgG1 isotype control (Biotin) (Biolegend, Catalogue:400-104, Clone: MOPC-21)

Validation

All monoclonal antibodies used were acquired from commercially available suppliers, and thus were validated by their manufacturers Details of the validation can be retrieved from the supplier name, catalogue number, clone and lot number provided above. In the lab, mAB titrations were performed to determine minimal working concentration for experimentation, and standardized for all pull-down immunoaffinity assays as described in the methods section.

Eukaryotic cell lines

Policy information about cell lines and Sex and Gender in Research

Cell line source(s)

All human ovarian cancer cell lines described in this study were obtained from ATCC (CaOV3, OV90, and ES2).
CaOV3 (ATCC, Catalogue: Caov-3, HTB-75)
OV90 (ATCC, Catalogue: CRL-3585)
ES2 (ATCC, Catalogue: CRL-1978)

The benign cell line, TIOSE4, was established by transfecting normal ovarian surface epithelium (NOSE) cells with hTERT.

Authentication

Cell lines were acquired commercially and authenticated by the supplier. Information about the validation can be retrieved based on the details provided above directly from the supplier. No other authentication was performed in the lab.

Mycoplasma contamination

Mycoplasma free.

Commonly misidentified lines
(See ICLAC register)

No commonly misidentified cell lines were used in this study.
