# Supplementary material for: Molecular fingerprinting of biological nanoparticles with a label-free optofluidic platform
Source: Nat Commun. 2024 May 15;15:4109. doi: 10.1038/s41467-024-48132-4 (PMC11096335; doi:10.1038/s41467-024-48132-4)
Supplement: Supplementary file 6 — Description of Additional Supplementary Files [file 41467_2024_48132_MOESM6_ESM.pdf]

Title: Supplementary Movie 1

Description: Following the on-chip surface functionalisation in real time: peptide assisted bilayer formation. The video shows a zoomed area corresponding to 31.2 μm x 31.2 μm of the bilayer formation process using 30 nm extruded liposomes. The total imaged area covers a total area of 0.2 mm^2. The data is recorded at 1 Hz and is represented with a contrast range of -0.04 to 0.03. Scale bar: 10 μm.

Title: Supplementary Movie 2

Description: Following the on-chip surface functionalisation in real time: liposome fusion bilayer formation. The video shows a zoomed area corresponding to 31.2 μm x 31.2 μm of the bilayer formation process using sonicated liposomes ranging in size from 100-3000 nm. The total imaged area covers a total area of 0.2 mm^2. The data were recorded at 1 Hz and are represented with a contrast range of -0.04 to 0.03. Scale bar: 10 μm.
