# Supplementary material for: Molecular fingerprinting of biological nanoparticles with a label-free optofluidic platform
Source: Nat Commun. 2024 May 15;15:4109. doi: 10.1038/s41467-024-48132-4 (PMC11096335; doi:10.1038/s41467-024-48132-4)
Supplement: Supplementary file 7 — Source data [file 41467_2024_48132_MOESM7_ESM.zip › FigureData/FigureS7.zip!/WB_summary_ES2/summary.pdf]

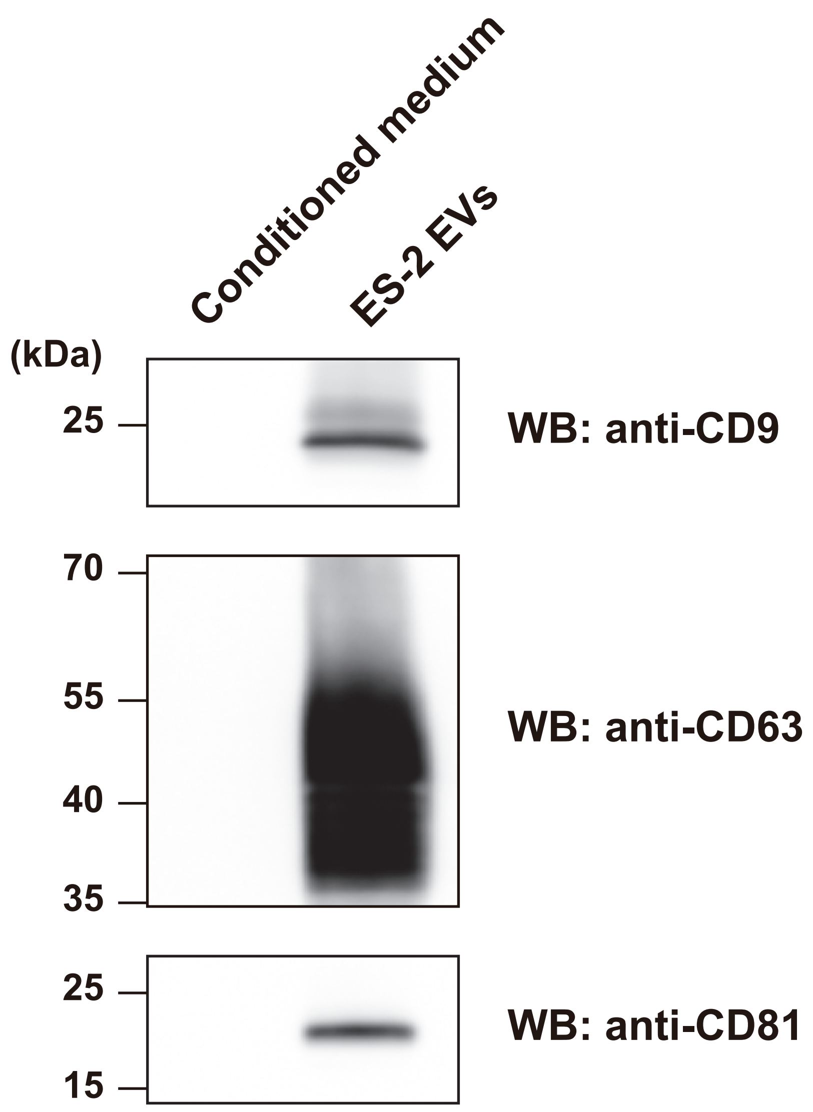

CD9: Biolegend, 312102, 1:500
CD63: Ancell, 215-820, 1:1000
CD81: Santa Cruz, sc-166029, 1:500
